# Ultrasonographic evaluation of the diaphragm in critically ill patients to predict invasive mechanical ventilation

**DOI:** 10.1186/s40560-023-00690-3

**Published:** 2023-09-19

**Authors:** Karn Suttapanit, Supawit Wongkrasunt, Sorravit Savatmongkorngul, Praphaphorn Supatanakij

**Affiliations:** Department of Emergency Medicine, Faculty of Medicine, Ramathibodi Hospital, Mahidol University, 270 Rama VI Rd., Ratchathewi, Bangkok, 10400 Thailand

**Keywords:** Diaphragmatic excursion, Point-of-care ultrasound, Diaphragmatic dysfunction, Critical illness

## Abstract

**Background:**

Diaphragm dysfunction is common in critically ill patients and associated with poorer outcomes. The function of the diaphragm can be evaluated at the bedside by measuring diaphragmatic excursion using ultrasonography. In this study, we investigated the ability of right-sided diaphragmatic excursion (RDE) to predict the need for invasive mechanical ventilation (IMV).

**Methods:**

Critically ill patients aged 18 years and older who presented to our emergency department between May 20, 2021 and May 19, 2022 and underwent measurement of RDE within 10 min of arrival were enrolled in this prospective study. The ability of RDE to predict the need for IMV was assessed by multivariable logistic regression and analysis of the area under the receiver-operating characteristic curve (AUROC).

**Results:**

A total of 314 patients were enrolled in the study; 113 (35.9%) of these patients required IMV. An increase of RDE value per each 0.1 cm was identified to be an independent predictor of IMV (adjusted odds ratio 0.08, 95% confidence interval [CI] 0.04–0.17, *p* < 0.001; AUROC 0.850, 95% CI 0.807–0.894). The RDE cutoff value was 1.2 cm (sensitivity 82.3%, 95% CI 74.0–88.8; specificity 78.1%, 95% CI 71.7–83.6). Time on a ventilator was significantly longer when the RDE was ≤ 1.2 cm (13 days [interquartile range 5, 27] versus 5 days [interquartile range 3, 8], *p* = 0.006).

**Conclusions:**

In this study, RDE had a good ability to predict the need for IMV in critically ill patients. The optimal RDE cutoff value was 1.2 cm. Its benefit in patient management requires further investigation.

**Supplementary Information:**

The online version contains supplementary material available at 10.1186/s40560-023-00690-3.

## Introduction

Critically ill patients who present in the emergency department (ED) often require respiratory support. Most of the evidence shows that non-invasive ventilation (NIV) and high-flow nasal cannula (HFNC) therapy reduce the likelihood of intubation in these patients [1–3]. However, delayed intubation is associated with high mortality. Therefore, close monitoring of vital signs and respiratory parameters is essential [1, 4–7].

The diaphragm is the main muscle involved in respiration. Diaphragm dysfunction is common in critically ill patients and is associated with worse outcomes, including requirement for invasive mechanical ventilation (IMV) and an increased mortality risk [8–11]. The function of the diaphragm can be evaluated by ultrasonography at the bedside in the intensive care unit (ICU) or ED to predict risks of intubation and re-intubation [11–13]. Measurement of diaphragmatic excursion by ultrasound is one of the methods that can be used to detect diaphragm dysfunction. In the ED, a few studies had utility diaphragmatic excursion measurement to predicting require IMV [14, 15]. A previous study showed that the decreased diaphragmatic excursion value in the ED has a good ability to detect paralysis of the diaphragm in patients with acute dyspnea and to predict failure of NIV in patients with acute exacerbation of chronic obstructive pulmonary disease [14]. However, the accuracy of measurement of diaphragmatic excursion in the ED and the cutoff value that predicts the need for IMV in critically ill patients remains to be determined [14, 15].

The aim of this study was to evaluate the ability of right-sided diaphragmatic excursion (RDE) to predict the need for IMV and to determine its optimal cutoff value.

## Methods

### Study design

This prospective single-center cohort study was performed at Ramathibodi Hospital, which is a tertiary teaching healthcare facility in Bangkok, Thailand. The study was approved by the Ethics Committee of Ramathibodi Hospital, Mahidol University (approval number COA MURA2021/403). Each study participant or a family member provided written informed consent before enrollment in the study.

### Patient selection process and eligibility criteria

Critically ill patients aged 18 years or older who presented to the ED between May 20, 2021 and May 19, 2022 were enrolled. In terms of critically ill patients, any condition of vital organ dysfunction who high risk of life threatening [16]. Critical illness in this study was defined as any of the following: a respiratory rate > 24 breaths per minute, use of accessory respiratory muscles, hypoxemia (pulse oxygen saturation < 90% or ratio of arterial oxygen partial pressure to a fraction of inspired oxygen (PF ratio) < 300), hypotension (systolic blood pressure (SBP) < 90 mmHg or mean arterial pressure (MAP) < 65 mmHg), and a Glasgow Coma Scale (GCS) score of < 15 [17, 18]. Patients with a neuromuscular disorder, those who underwent endotracheal intubation within 60 min of arrival, those with traumatic injury, and those with a do-not-attempt intubation order were excluded.

### Data measurements

Data were collected on patient background characteristics (sex, age, and body mass index), comorbidities, Charlson Comorbidity Index, heart rate, blood pressure, respiratory rate, blood oxygen saturation, PF ratio, GSC score, blood pH, partial pressure of arterial oxygen (PaO_2_), partial pressure of arterial carbon dioxide (PaCO_2_), bicarbonate level, blood lactate, serum creatinine, Sequential Organ Failure Assessment (SOFA) score, final diagnosis, and diaphragmatic excursion at the time of enrollment in the ED.

The fraction of inspired oxygen (FiO_2_) to use in the calculation PF ratio in conventional oxygen therapy (COT) such as nasal cannula and non-rebreathing mask with reservoir bag was estimated by 3% formula (21% + oxygen flow rate in L/min × 3) [19, 20]. In a previous study in HFNC with flow rate > 30 LPM in closed, delivered FiO_2_ was closed to measured FiO_2_ [21]. Hence, the delivered FiO_2_ in HFNC and NIV was collected from the setting to calculate the PF ratio. The decision to use COT, HFNC and NIV was dependent on the physician’s judgment.

Diaphragmatic excursion was measured by ultrasonography within 10 min of arrival in the ED by experienced emergency physicians, each of whom had performed more than 50 such measurements. All measurements were obtained on the right side during spontaneous tidal breathing (quiet breathing) without non-invasive respiratory support (NIV or HFNC) in a semi-recumbent position. The transducer was placed in the right subcostal area between the midclavicular and anterior axillary lines. The angle of ultrasound tracing is possible cranially and perpendicular to the dome of the diaphragm. Diaphragmatic excursion was measured in M-mode. The RDE was defined as the distance between the value of the diaphragm dome in end-inspiratory and end-expiratory (Additional file 1: S1) [8], which was measured using a Xario 100G ultrasound machine (Canon Medical Systems USA Inc., Tustin, CA, USA) with a 1.8–4.8-MHz sector probe. The average of three measurements was recorded. The emergency physician who performed the ultrasound was not involved in decision-making regarding IMV.

The requirement for IMV was based on the following: hypoxic respiratory failure defined by hypoxemia with PF ratio < 150, respiratory rate > 35 breaths per minute, significant accessory respiratory used after management with non-invasive respiratory support, respiratory acidosis defined by pH < 7.35 and PaCO_2_ > 45 mmHg while receiving non-invasive respiratory support, persistent hemodynamic instability after optimization of fluids and vasoactive agents, and need for intubation to protect the airway [22].

The primary outcome was the ability of RDE to predict the requirement for IMV within 48 h and its optimal cutoff value. Secondary outcomes were the associations of RDE with durations of IMV and mortality.

### Calculation of sample size

The minimum total sample size required to detect an area under the receiver-operating characteristic curve (AUROC) value of 0.80 with an effect size of 0.10, the 95% confidence interval (CI), and a power of 80% was calculated to be 184 [23].

### Statistical analysis

Patients were compared according to whether or not they required IMV. Categorical variables are expressed as the number (percentage) and continuous variables as the mean ± standard deviation if normally distributed. Variables with a skewed distribution are shown as the median (interquartile range [IQR]). Categorical variables were compared using the chi-squared test, and continuous variables using the unpaired *t* test or Mann–Whitney *U* test as appropriate.

Multivariable logistic regression was used to identify whether RDE was associated with the requirement for IMV. Important factors at risk in intubation, such as blood pH < 7.30, GCS score < 15, hypotension, respiratory rate > 30 per minute, and PF ratio < 200, and factors with *p* value < 0.100 in univariable logistic regression, were selected for regression. In multivariable logistic regression, multicollinearity was checked by a variance inflation factor (VIF), factors of which VIF > 2.5 will be removed. The AUROC method was used to assess the predictive performance of RDE. The algorithm of DeLong was used to compare AUROC in each variable. Sensitivity and specificity values were used to determine the optimal cutoff value for RDE. The probability of requiring IMV within 48 h according to the cutoff value was determined using Kaplan–Meier survival curves. The statistical analysis was performed using STATA version 16.1 (StataCorp LLC, College Station, TX, USA). A *p* value of < 0.05 was considered statistically significant.

## Results

RDE was measured by ultrasound in 340 patients during the study period. Twenty-six patients were excluded (do-not-attempt intubation order, *n* = 14; required IMV within 60 min, *n* = 10); neuromuscular disorder, *n* = 2), leaving 314 patients for inclusion in the final analysis.

Two hundred and nineteen of the 314 patients received non-invasive respiratory support, 163 received NIV, 49 received HFNC, and seven received both NIV and HFNC (Table 1). One hundred and thirteen patients (35.9%) required IMV; 67 (59.3%) required IMV within 6 h, and 88 (77.9%) within 24 h of ultrasound evaluation. The median RDE value was 0.90 cm (IQR 0.76, 1.10) in patients who required IMV within 48 h and 1.80 cm (IQR 1.30, 2.40) in those who did not. In sex, the median RDE values were 0.9 cm (IQR 0.70, 1.10) and 1.0 cm (IQR 0.80, 1.20) in female and male patients who required IMV within 48 h, respectively, significantly different (*p* = 0.039). Moreover, the median RDE in patients who received non-invasive respiratory support was 0.90 cm (IQR 0.70, 1.10) in intubated patients and 1.70 cm (IQR 1.20, 2.40) in those who did not intubate (Additional file 2: S2).

**Table 1:** Patient demographic and clinical characteristics

Variable	Intubated patients (*n* = 113)	Non-intubated patients (*n* = 201)	*p* value
Sex, male, *n* (%)	68 (60.2)	101 (50.2)	0.091
Age (years), median [IQR]	77 [67, 83]	73 [65, 85]	0.603
BMI (kg/m^2^), mean ± SD	22.7 ± 5.5	22.9 ± 5.8	0.678
Charlson Comorbidity Index, median [IQR]	3 [1, 5]	3 [1, 5]	0.968
Comorbidities, *n* (%)			
COPD	22 (19.5)	42 (20.9)	0.764
Chronic renal disease stage 4 or 5	13 (11.5)	37 (18.4)	0.127
History of myocardial infarction	31 (27.4)	47 (23.4)	0.426
Heart failure	32 (28.3)	71 (35.3)	0.205
History of a cerebrovascular event	32 (28.3)	54 (26.9)	0.782
Dementia	18 (15.9)	24 (11.9)	0.320
Post-COVID-19 status	18 (15.9)	25 (12.4)	0.388
Vital signs			
SBP, mmHg, mean ± SD	126 ± 35	132 ± 34	0.137
MAP, mmHg, mean ± SD	88 ± 23	92 ± 22	0.072
Hypotension (SBP ≤ 90 or MAP ≤ 65 mmHg), *n* (%)	15 (13.3)	24 (11.9)	0.731
Heart rate, bpm, mean ± SD	102 ± 23	98 ± 23	0.216
Respiratory rate, per minute, median [IQR]	28 [24, 32]	26 [24, 30]	0.014
Respiratory rate > 30 per minute, *n* (%)	33 (29.2)	42 (20.1)	0.098
SpO_2_, (%), median [IQR]	94 [90, 98]	96 [92, 99]	0.006
Glasgow Coma Scale score, median [IQR]	15 [13, 15]	15 [15]	< 0.001
Glasgow Coma Scale score < 15, *n* (%)	41 (36.3)	33 (16.4)	0.002
Blood gas analysis			
pH, mean ± SD	7.37 ± 0.11	7.41 ± 0.08	0.001
PaO_2_, mmHg, median [IQR]	125 [84, 18]	130 [82, 172]	0.362
PaCO_2_, mmHg, median [IQR]	33 [27, 42]	33 [29, 40]	0.910
PF ratio, mean ± SD	377.77 ± 137.47	391.87 ± 135.20	0.379
Bicarbonate, mEq/L, mean ± SD	19.9 ± 6.1	21.6 ± 4.8	0.007
Blood lactate, mmol/L, median [IQR]	1.7 [0.8, 3.3]	1.8 [1.0, 2.8]	0.717
Serum creatinine, mg/dL, median [IQR]	1.18 [0.79, 1.72]	1.14 [0.82, 1.82]	0.848
SOFA score, median [IQR]	3 [2, 5]	2 [1, 4]	0.001
Non-invasive respiratory support, *n* (%)	88 (77.9)	131 (65.2)	0.019
Final diagnosis, *n* (%)			
Respiratory infection	63 (55.8)	73 (36.3)	0.001
Sepsis	76 (67.3)	73 (36.3)	< 0.001
Acute exacerbation of COPD	20 (17.7)	37 (18.4)	0.876
Asthma attack	4 (3.5)	10 (5.0)	0.555
Cardiogenic pulmonary edema	22 (19.5)	61 (30.3)	0.036
COVID-19 pneumonia	17 (15.0)	7 (3.5)	0.050
Diaphragmatic excursion, cm, median [IQR]	0.9 [0.76, 1.10]	1.8 [1.30, 2.40]	< 0.001

*BMI* body mass index, *COPD* chronic obstructive pulmonary disease, *COVID-19* coronavirus disease 2019, *IQR* interquartile range, *PaO*_*2*_ partial pressure of arterial oxygen, *PaCO*_*2*_ partial pressure of arterial carbon dioxide, *PF ratio* ratio of the partial pressure of arterial oxygen to inspired oxygen fraction, *SD* standard deviation, *SOFA* Sequential Organ Failure Assessment, *SpO*_*2*_ blood oxygen saturation level

Multiple logistic regression showed that the following five variables were significant independent predictors of the probability of requiring IMV within 48 h: increase RDE per 0.1 cm (adjusted odds ratio [aOR] 0.08, 95% CI 0.04–0.17, *p* < 0.001), PaO_2_ < 200 (aOR 0.26, 95% CI 0.07–0.96, *p* = 0.044), blood pH < 7.30 (aOR 3.79, 95% CI 1.36–10.57, *p* = 0.011), respiratory tract infection (aOR 2.31, 95% CI 1.12–4.77, *p* = 0.024), and sepsis (aOR 2.37, 95% CI 1.07–5.25, *p* = 0.033) (Table 2).

**Table 2:** Univariable and multivariable logistic regression of factors associated with intubation within 48 h of critically ill patients in the emergency department

Variable	Odds ratio (95% CI)	*p* value	Adjusted odds ratio (95% CI)	*p* value
Sex, male	1.50 (0.94–2.38)	0.091	1.50 (0.77–2.94)	0.237
Hypotension (SBP ≤ 90 or MAP ≤ 65 mmHg)	1.13 (0.57–2.25)	0.731	0.61 (0.21–1.75)	0.362
Respiratory rate > 30 per minute, *n* (%)	1.56 (0.920–2.65)	0.099	1.05 (0.56–2.26)	0.892
Glasgow Coma Scale score < 15	2.90 (1.70–4.95)	< 0.001	1.63 (0.72–3.66)	0.236
pH < 7.30	4.91 (2.31–10.42)	< 0.001	3.79 (1.36–10.57)	0.011
PF ratio < 200	0.76 (0.30–1.92)	0.565	0.26 (0.07–0.96)	0.044
Bicarbonate	0.94 (0.89–0.98)	0.007	0.99 (0.92–1.06)	0.678
Blood lactate ≥ 4 mmol/L	2.32 (1.19–4.50)	0.013	2.01 (0.72–5.61)	0.182
SOFA score	1.17 (1.06–1.29)	0.002	1.06 (0.88–1.29)	0.528
Non-invasive respiratory support	1.88 (1.11–3.20)	0.020	2.02 (0.93–4.33)	0.073
Final diagnosis				
Respiratory infection	2.21 (1.38–3.53)	0.001	2.31 (1.12–4.77)	0.024
Sepsis	3.60 (2.21–5.86)	< 0.001	2.37 (1.07–5.25)	0.033
Cardiogenic pulmonary edema	0.55 (0.32–0.97)	0.036	1.02 (0.46–2.26)	0.963
COVID-19 pneumonia	2.05 (0.99–4.23)	0.053	1.70 (0.59–4.91)	0.324
Diaphragmatic excursion (increase per 0.1 cm)	0.08 (0.05–0.16)	< 0.001	0.08 (0.04–0.17)	< 0.001

*BMI* body mass index, *CI* confidence interval, *COPD* chronic obstructive pulmonary disease, *COVID-19* coronavirus disease 2019, *MAP* mean arterial pressure, *PaCO*_*2*_ partial pressure of arterial carbon dioxide, *PaO*_*2*_ partial pressure of arterial oxygen, *PF ratio* ratio of the partial pressure of arterial oxygen to inspired oxygen fraction, *SBP* systolic blood pressure, *SOFA* Sequential Organ Failure Assessment, *SpO*_*2*_ blood oxygen saturation level

### The ability of RDE to predict required for IMV

**Fig. 1:**
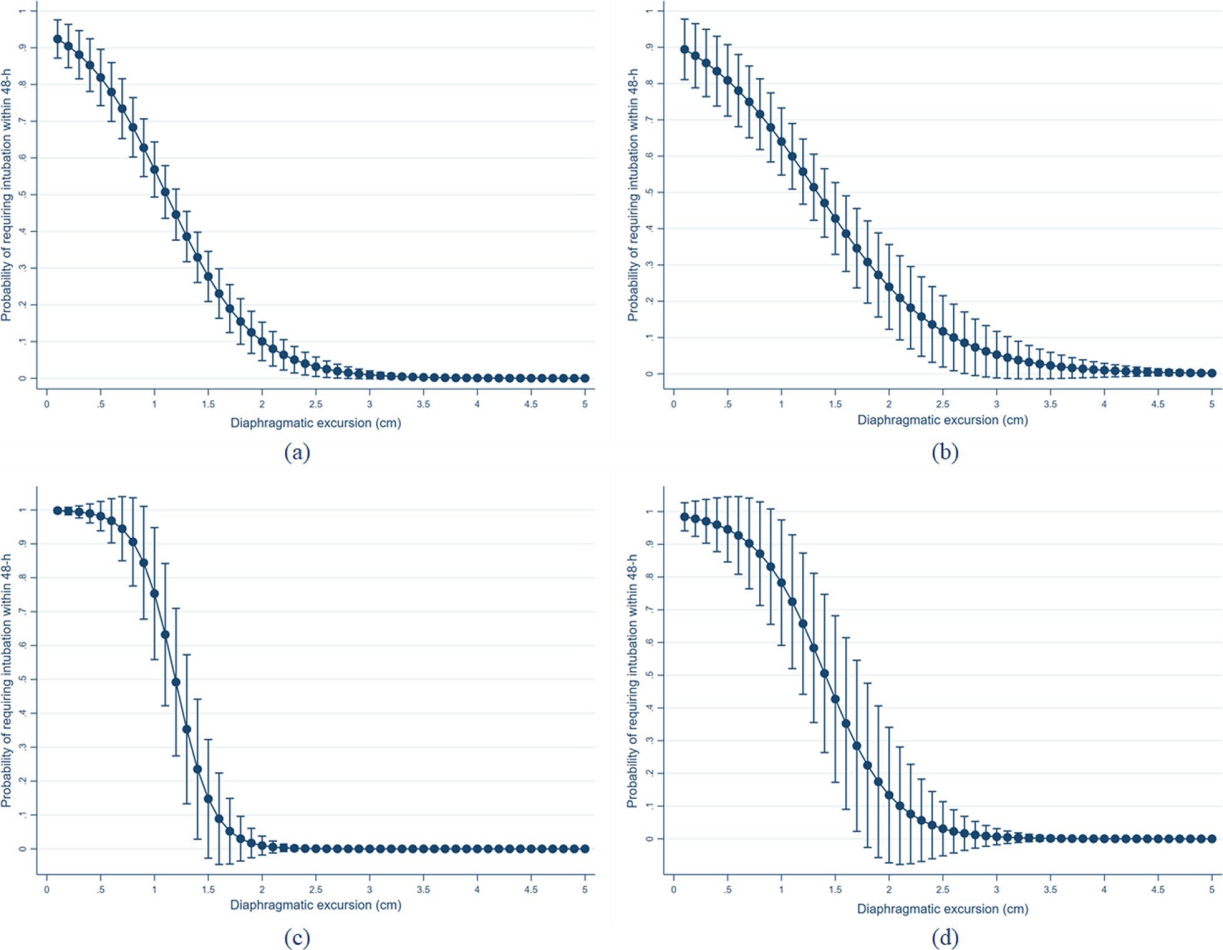
HYPERLINK "sps:id::fig1||locator::gr1||MediaObject::0"Probability of requiring intubation within 48 h in each value of right-sided diaphragmatic excursion **a** total populations, **b** sepsis, **c** acute exacerbation of chronic obstructive pulmonary disease and **d** coronavirus disease 2019

The AUROC for the ability of RDE to predict the requirement for IMV within 48 h was 0.850 (95% CI 0.807–0.894) and had better accuracy than blood pH (AUROC 0.601, 95% CI 0.533–0.669), and the SOFA score (AUROC 0.613, 95% CI 0.549–0.676). This finding was statistically significant between RDE and blood pH (*p* < 0.001), and between RDE and SOFA score (*p* < 001). In addition, the probability of requiring intubation within 48 h was increased in decreased RDE values (Fig. 1). Moreover, the association between RDE and non-invasive respiratory support was OR 0.76 (95% CI 0.60–0.96, *p* = 0.022), and AUROC for predicting requiring non-respiratory support was 0.589 (95% CI 0.521–0.657).

**Fig. 2:**
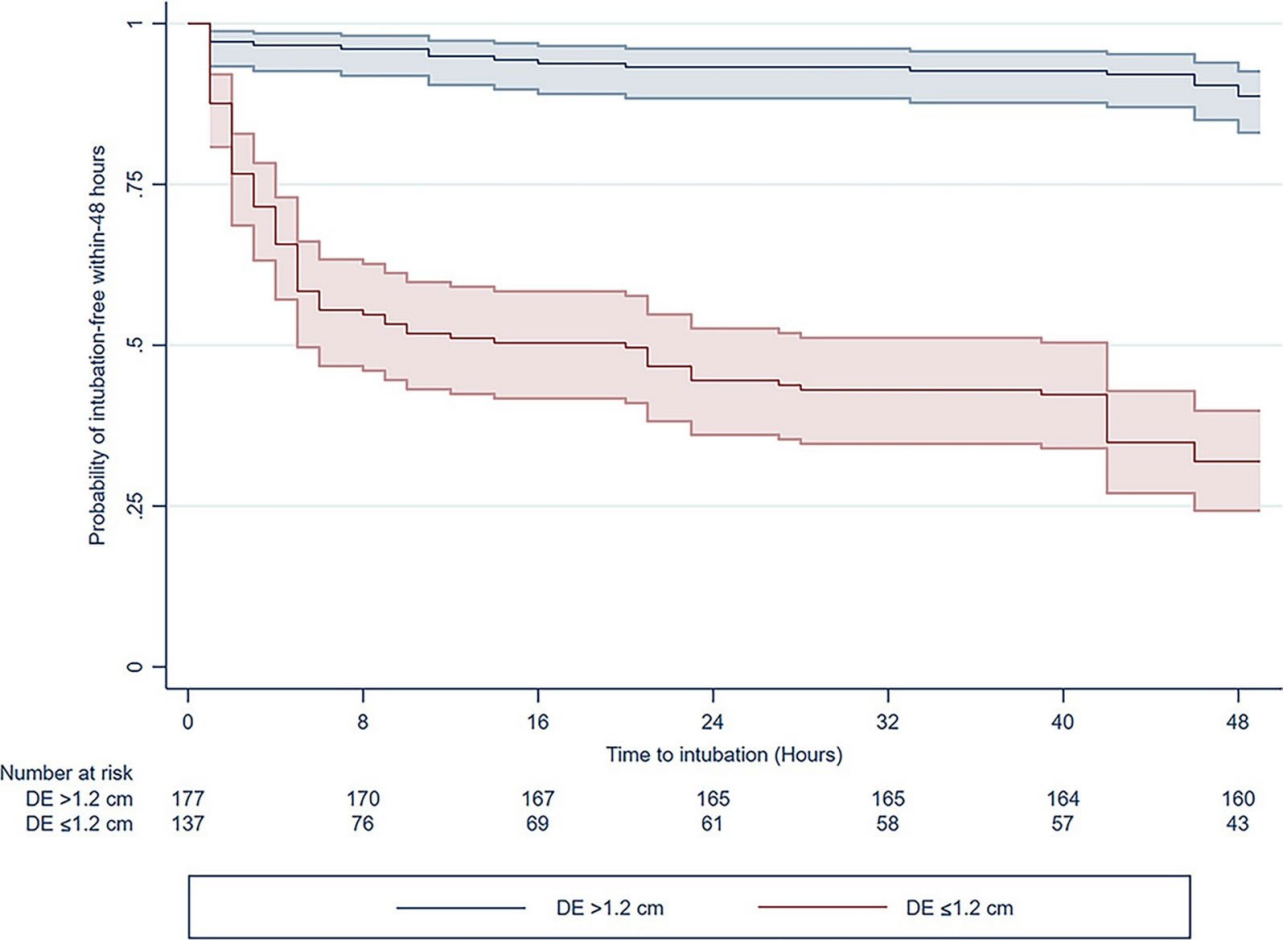
HYPERLINK "sps:id::fig2||locator::gr2||MediaObject::0"Kaplan–Meier survival curves for the probability of intubation-free within-48 h according to the cutoff value of the right-sided diaphragmatic excursion

The RDE cutoff value was 1.20 cm with a sensitivity of 82.3% (95% CI 74.0–88.8) and a specificity of 78.1% (95% CI 71.7–83.6) (Table 3). The Kaplan–Meier curves for the probability of intubation-free within 48 h are shown according to the cutoff value in Fig. 2. In addition, 110 (80.3%) of 137 patients with RDE ≤ 1.20 cm required non-invasive respiratory support, and 75 (68.1%) patients required IMV within 48 h.

**Table 3:** Predictive performance of diaphragmatic excursion in intubation within 48 h

Diaphragmatic excursion (cm)	Sensitivity	Specificity	PPV	NPV	Predicted probability
Cohort (*N* = 314), AUROC 0.850 (95% CI 0.807–0.894)					
≥ 2.00	–	–	–	–	3.4% (− 0.4, 7.3)
1.60–1.99	97.3% (92.4–99.4)	41.8% (34.9–48.9)	48.5% (41.8–55.2)	96.6% (90.3–99.3)	12.8% (3.2–22.3)
1.21–1.59	92.2% (85.4–96.3)	62.2% (55.1–68.9)	57.8% (50.2–65.1)	93.3% (87.6–96.9)	25.6% (12.5–38.6)
0.91–1.20	82.3% (74.0–88.8)	78.1% (71.7–83.6)	67.9% (59.4–75.6)	88.7% (83.1–93.0)	58.5% (45.2–71.8)
≤ 0.90	54.9% (45.2–64.2)	89.1% (83.9–93.0)	73.8% (63.1–82.8)	77.8% (71.9–83.0)	73.8% (64.4–83.2)

*AUROC* area under the receiver-operating characteristic curve, *NPV* negative predictive value, *PPV* positive predictive value

In addition, the AUROCs for the ability of the RDE to predict the requirement for IMV within 6 h and 24 h were 0.841 (95% CI 0.794–0.889) and 0.854 (95% CI 0.811–0.899), respectively. The median RDE value was 0.80 cm (IQR 0.70, 1.10) in patients who required IMV within 6 h and 0.90 cm (IQR 0.70, 1.10) in patients who required IMV within 24 h.

**Fig. 3:**
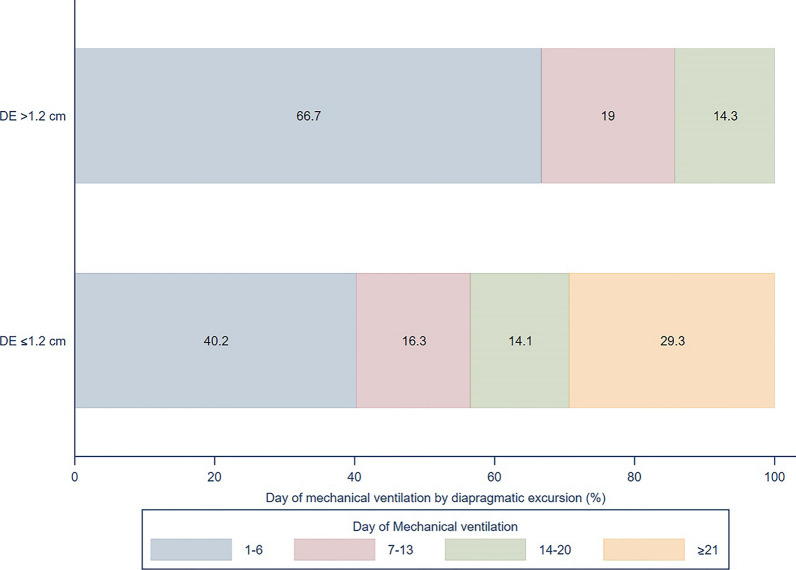
HYPERLINK "sps:id::fig3||locator::gr3||MediaObject::0"Probability of number of days on mechanical ventilation according to the right-sided diaphragmatic excursion cutoff value

Ventilation time was significantly longer when the RDE was ≤ 1.2 cm than when it was > 1.2 cm (13 days [IQR 5, 27] versus 5 days [IQR 3, 8], *p* = 0.006). The probability of mechanical ventilation according to the RDE threshold is shown in Fig. 3. Moreover, RDE ≤ 1.2 cm was an independent risk factor for mortality (aOR 2.99, 95% CI 1.32–6.79, *p* = 0.009), including when adjusted by SOFA score (aOR 1.17, 95% CI 1.02–1.34, *p* = 0.020), blood lactate ≥ 4 mmol/L (aOR 1.13, 95% CI 0.46–2.81, *p* value = 0.788), blood pH < 7.30 (aOR 0.45, 95% CI 0.15–1.34, *p* = 0.152), and receiving IMV (aOR 2.94, 95% CI 1.34–6.42, *p* = 0.007).

On subgroup analysis according to the final diagnosis, the AUROC was 0.893 (95% CI 0.782–0.999) for presence of acute exacerbation of chronic obstructive pulmonary disease (AECOPD), 0.830 (95% CI 0.779–0.882) for non-AECOPD, 0.792 (95% CI 0.714–0.869) for sepsis, and 0.893 (95% CI 0.782–0.999) for coronavirus disease 2019 (COVID-19) pneumonia (Additional file 3: S3).

## Discussion

In this study, the RDE had a good ability to predict the need for IMV within 48 h in critically ill patients in ED and was better than the ratio of oxygen saturation to fraction of inspired oxygen and the SOFA score. Furthermore, a low RDE value was associated with longer IMV and increased mortality risk.

Point-of-care ultrasound (POCUS) has been found to be useful in the ICU and ED for guiding resuscitation of critically ill patients, such as fluid responsiveness, and the bedside lung ultrasound in emergency (BLUE) protocol for immediate diagnosis of respiratory failure [24–26]. Critical illness has been associated with diaphragm dysfunction that causes respiratory failure and difficulty in weaning from mechanical ventilation and with mortality [8, 9, 27]. Transdiaphragmatic pressure (Pdi) is used to measure diaphragmatic force; however, the gold standard of Pdi measurement is magnetic stimulation of phrenic nerves, which was unsuitable in ED [9]. Therefore, there is a need for evaluation of the ability of examination of the diaphragm by ultrasound to detect diaphragm dysfunction and predict the need for IMV.

The diaphragm ultrasound can assess diaphragm function by diaphragmatic excursion and diaphragmatic thickness fraction. In this study, we used the RDE, which is simpler to measure and more rapidly obtained than the diaphragmatic thickness fraction when assessing the function of the diaphragm and has demonstrated good reliability [27–29]. The diaphragm action is qualified for shortening and force generation, which are measured by volume change and inspiratory pressure, respectively. The relation between diaphragmatic excursion and tidal volume and inspiratory capacity (IC) in spontaneous breathing at rest and exercise in healthy was a linear correlation [30, 31]. Moreover, in COPD, inspiratory capacity was reduced compared to volunteers who had no chronic disease, and decreasing of the diaphragmatic excursion was associated with a reduced inspiratory capacity [32]. In a previous study, diaphragmatic excursion has been a reliable tool as fluoroscopy for measuring diaphragm contractile activity [33]. In spontaneous breathing, diaphragmatic excursion correlated well with Pdi and esophageal pressure in intubated patients with zero pressure [34].

Previous studies have shown that diaphragmatic excursion has poor accuracy when used to predict the need for use of a mechanical ventilator. Clément et al. showed that diaphragmatic excursion could not predict the need for NIV or IMV in patients with acute dyspnea in the ED [15]. A similar study by Barbariol et al. found that diaphragmatic excursion had a poor ability to predict failure of NIV in patients with acute hypoxic respiratory failure in the ICU [35]. These findings are in contrast with those of our present study, in which the RDE showed a good ability to predict IMV. Possible reasons for this inconsistency are that our study population included patients with shock or a change in mental status and different criteria for intubation. Furthermore, we found that the RDE could predict IMV regardless of whether or not the patient had AECOPD, especially when associated with COVID-19 pneumonia.

The diaphragmatic dysfunction in critical conditions was related to worsening outcomes. Previous studies have identified mechanical ventilation use, malnutrition, use of corticosteroids, inflammation, releases of cytokines, and mitochondrial impairment as causes of diaphragm dysfunction in critical illness due to the catabolic process occurring in the diaphragm and other respiratory muscles [8, 36, 37]. Many studies show that sepsis is one of the risks of diaphragmatic dysfunction. Increasing metabolic demands and inflammation in sepsis increased respiratory drive and effort. However, in animal studies, group B streptococcal sepsis in young piglets was associated decline in diaphragmatic contractility and tidal volume within 2 h [38], and endotoxin administration produced a reduction in diaphragmatic force generation in hamsters [39]. Similarly, other experimental studies in animals exposed to endotoxin showed increased ventilation in an early, then fall and decreased diaphragmatic function [40–42]. Chen et al. found that diaphragmatic thickening fraction and diaphragmatic excursion were significantly lower in sepsis with SOFA > 5 than in controls [43]. In ICU, diaphragm volume and strength were lower in sepsis when compared with non-septic. Moreover, diaphragm strength was correlated with diaphragm volume [44]. Systemic inflammation in sepsis increased diaphragm weakness and susceptibility to injury. In addition, diaphragm dysfunction may develop within 4 h of sepsis. Therefore, diaphragmatic evaluation may consider monitoring to choose ventilatory support to prevent worsening outcomes in delayed intubation and diaphragmatic weakness in mechanical ventilation [8, 22]. A previous study evaluated the performance of diaphragmatic function was found low diaphragmatic excursion values associated need for IMV, prolonged IMV, and mortality in sepsis [45]. Similarly, this study found RDE was a good performance to predict IMV within 48 h and mortality. In our population, respiratory rate was high, and accessory muscle used that indicated high respiratory effort was included in the inclusion criteria. However, low RDE was associated with requiring IMV, which results similar results to previous study [40–42, 45]. Therefore, the evidence of diaphragmatic ultrasound needs evaluation and validation in requiring intubation in sepsis.

In COPD, expiratory flow is limited by airway narrowing resulting from chronic inflammation and mucus plugging, causing to required prolonged time to exhale volume in the lung. In critically ill, almost increased minute ventilation from increased tidal volume and respiratory rate that were increasing in end-expiratory lung volume and reduced IC. In COPD with limited IC, increasing the minute ventilation by increased respiratory rate cause of dynamic hyperinflation by insufficient expiratory time and causes hypercapnic respiratory failure, especially in AECOPD. In addition, the work of breathing increased during hyperinflation and resulting in diaphragmatic dysfunction [46]. Previous studies show diaphragmatic dysfunction was associated with required IMV [10, 12, 14]. Diaphragmatic thickening fraction was shown to correlate with Pdi during the sniff maneuver and accurately identified risks of NIV failure in AECOPD [12]. However, this study did not measure the diaphragmatic thickening fraction. Diaphragmatic excursion in COPD with acute hypercapnic respiratory failure could predict NIV failure more accurately than arterial pH and PaCO_2_ [14], similar to our study. This result was in line with previous findings that diaphragmatic excursion was correlated with inspiratory capacity and tidal volume [30–32]; in spontaneous breathing that result presumed the low value of diaphragmatic excursion was associated with decreased IC due to dynamic hyperinflation and caused requiring IMV in AECOPD.

In COVID-19, the theory of diaphragmatic dysfunction was related to critical illness myopathy, cytokine storm, ventilator-induced diaphragmatic dysfunction, and directly viral infiltration via expression of the angiotensin-converting enzyme 2 receptor [36, 37, 47]. Other studies have also found associations between diaphragm dysfunction with the requirement for IMV and adverse outcomes in patients with severe COVID-19 pneumonia [48, 49] that support the results of our study. However, the sample size in our study on COVID-19 pneumonia was small. Therefore, clinical application in diaphragmatic ultrasound should be assessed in further study.

The normal diaphragmatic excursion value in quiet breathing is about 2 cm in the general population and 1.0–1.4 cm in critically ill patients in a semi-recumbent position [27, 29, 50]. In our study, the median RDE value was 0.9 cm in critically ill patients requiring IMV and 1.8 cm in their counterparts who did not; both these values are lower than those in the general population. Moreover, our study showed different diaphragmatic excursion between gender, since the excursion in males was displace greater than in females, that result supports previous studies [29, 50]. Several other studies have shown an association between a low value of diaphragmatic excursion value and requirements for IMV [15, 35, 42]. Many studies have shown various cutoff values of diaphragmatic excursion to be associated with adverse events [27, 29]. Therefore, our study showed sensitivity, specificity, and probability in predicting requiring IMV in each range of RDE for physician decisions. This study identified an RDE of 1.2 cm as the cutoff value below which there was a high probability of requiring IMV and an RDE of ≥ 2.0 cm to be the value above which the probability was low.

Motion of the diaphragm, indicated by the diaphragmatic thickness fraction and excursion, has been assessed as a prognostic factor in weaning from mechanical ventilation in the ICU. Patients with a diaphragmatic excursion or diaphragmatic thickness fraction lower than the threshold were found to have a prolonged period of IMV, which was a predictor of failure to extubate [8, 9]. In our study, an RDE ≤ 1.2 cm was similarly associated with a significantly prolonged period of IMV.

In view of our results, we believe that the RDE could be useful to implement in POCUS for assessment of the risk of failure of non-invasive respiratory support and identifying patients at risk who require close monitoring. For example, patients who present in the ED with acute respiratory distress and an RDE ≤ 1.2 cm could be admitted to ICU or a respiratory care unit for close monitoring and early intubation for IMV to prevent adverse conditions, such as mortality. In contrast, patients with an RDE > 2 cm could safely receive non-invasive respiratory support and routine care outside the ICU. Furthermore, monitoring the RDE during the weaning process could help to predict the outcomes of weaning from IMV. Using diaphragmatic excursion with clinical parameters that indicated intubation criteria could benefit the decision for early intubation to prevent adverse outcomes. However, integrating diaphragmatic excursion into POCUS to guide the resuscitation setting requires further studies to confirm the cutoff value and the benefit of implementing care.

This study has several limitations. First, it was performed in the ED of a single tertiary care center, which limits the generalizability of its findings. However, the sample size was adequate for assessments of the primary outcome. Second, we used the average of the RDE values obtained on ultrasound by a single emergency physician for each patient; therefore, the intra-rater and inter-rater correlations were not analyzed. However, all the emergency physicians who measured the diaphragmatic excursion had already performed the procedure more than 50 times, and previous research has shown that measurement of diaphragmatic excursion is an easy skill to master with a steep learning curve and good reliability [27, 28]. Third, we measured only RDE and did not include the diaphragmatic thickness fraction, which has been shown to be associated with respiratory effort in patients receiving positive pressure ventilation [27, 51]. Nevertheless, RDE was measured during spontaneous breathing, which has been reported to produce reliable results [27, 29]. Fourth, ultrasound was performed only once and not repeated after resuscitation. Finally, confounding factors, such as pharmacologic treatment, setting, and parameters of non-invasive respiratory support, were not collected in this study. Finally, the RDE value was unblinded to physicians who decided on intubation; however, the emergency physician who performed the ultrasound was not involved in deciding on intubation. Further studies are needed to validate our cutoff value in multi-center studies and determine the diaphragmatic excursion and the thickness fraction or a variation of diaphragmatic parameters should be implemented in POCUS to predict worsening outcomes.

## Conclusions

In this study, the RDE had a good ability to predict the need for IMV in critically ill patients. A low diaphragmatic excursion value was associated with a need for IMV; the cutoff value was 1.2 cm. Patients with an RDE below the cutoff value required a longer period of IMV. The benefit of inclusion of the RDE in POCUS should be assessed further.

### Supplementary Information

**Additional file 1: S1.** (a) The transducer (sector probe) was placed in the right subcostal area between the midclavicular and anterior axillary lines. (b) The angle of ultrasound tracing is possible to the diaphragmatic dome. The diaphragmatic excursion was measured in M-mode. The right diaphragmatic excursion (red dash) was measured as the distance between the value of the diaphragm dome in end-inspiration and end-expiration (green dash).

**Additional file 2: S2.** Comparison of right-sided diaphragmatic excursion between the non-intubated group and the intubated group in received and not received non-invasive respiratory support.

**Additional file 3: S3.** Predictive performance of diaphragmatic excursion in the emergency department according to the final diagnosis.

## Data Availability

All data generated or analyzed during this study are included in this published article and its Additional files 1, 2, and 3.

## Abbreviations

AECOPD: Acute exacerbation of chronic obstructive pulmonary disease
aOR: Adjusted odds ratio
AUROC: Area under the receiver-operating characteristic curve
BLUE: Bedside lung ultrasound in emergency
CI: Confidence interval
COT: Conventional oxygen therapy
ED: Emergency department
FiO_2_: Fraction of inspired oxygen
GCS: Glasgow Coma Scale
HFNC: High-flow nasal cannula
IC: Inspiratory capacity
ICU: Intensive care unit
IMV: Invasive mechanical ventilation
IQR: Interquartile range
MAP: Mean arterial pressure
NIV: Non-invasive ventilation
PaCO_2_: Partial pressure of arterial carbon dioxide
PaO_2_: Partial pressure of arterial oxygen
Pdi: Transdiaphragmatic pressure
PFratio: Ratio of arterial oxygen partial pressure to a fraction of inspired oxygen
POCUS: Point-of-care ultrasound
RDE: Right-sided diaphragmatic excursion
SBP: Systolic blood pressure
SOFA: Sequential Organ Failure Assessment
VIF: Variance inflation factor
