# Supplementary material for: Ultrasonographic evaluation of the diaphragm in critically ill patients to predict invasive mechanical ventilation
Source: J Intensive Care. 2023 Sep 19;11:40. doi: 10.1186/s40560-023-00690-3 (PMC10507830; doi:10.1186/s40560-023-00690-3)
Supplement: Supplementary file 1 — Additional file 1: S1. (a) The transducer (sector probe) was placed in the right subcostal area between the midclavicular and anterior axillary lines. (b) The angle of ultrasound tracing is possible to the diaphragmatic dome. The diaphragmatic excursion was measured in M-mode. The right diaphragmatic excursion (red dash) was measured as the distance between the value of the diaphragm dome in end-inspiration and end-expiration (green dash). [file 40560_2023_690_MOESM1_ESM.docx]

**
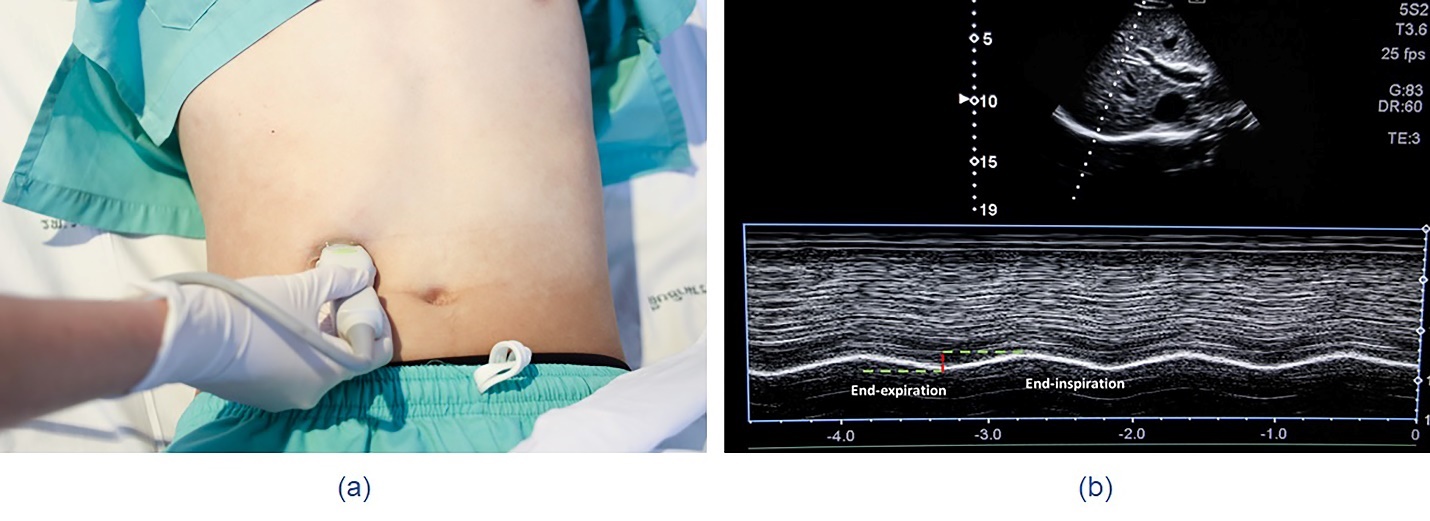
**

**Additional File S1.** (a) The transducer (sector probe) was placed in the right subcostal area between the midclavicular and anterior axillary lines. (b) The angle of ultrasound tracing is possible to the diaphragmatic dome. The diaphragmatic excursion was measured in M-mode. The right diaphragmatic excursion (red dash) was measured as the distance between the value of the diaphragm dome in end-inspiration and end-expiration (green dash).
