# Supplementary material for: Ultrasonographic evaluation of the diaphragm in critically ill patients to predict invasive mechanical ventilation
Source: J Intensive Care. 2023 Sep 19;11:40. doi: 10.1186/s40560-023-00690-3 (PMC10507830; doi:10.1186/s40560-023-00690-3)
Supplement: Supplementary file 2 — Additional file 2: S2. Comparison of right-sided diaphragmatic excursion between the non-intubated group and the intubated group in received and not received non-invasive respiratory support. [file 40560_2023_690_MOESM2_ESM.docx]

**
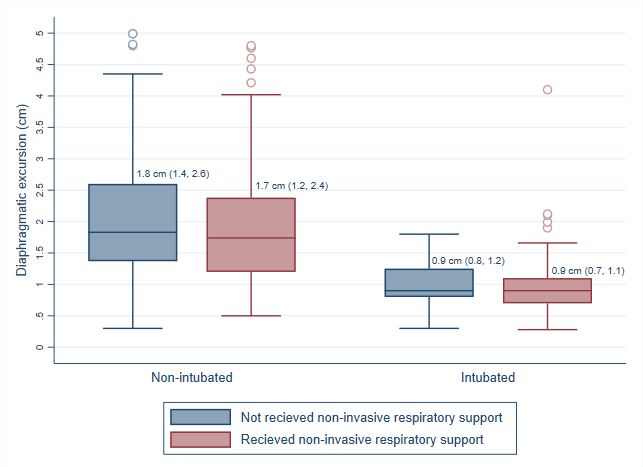
**

**Additional File S2.** Comparison of right-sided diaphragmatic excursion between the non-intubated group and the intubated group in received and not received non-invasive respiratory support.
