# Supplementary material for: Ultrasonographic evaluation of the diaphragm in critically ill patients to predict invasive mechanical ventilation
Source: J Intensive Care. 2023 Sep 19;11:40. doi: 10.1186/s40560-023-00690-3 (PMC10507830; doi:10.1186/s40560-023-00690-3)
Supplement: Supplementary file 3 — Additional file 3: S3. Predictive performance of diaphragmatic excursion in the emergency department according to the final diagnosis. [file 40560_2023_690_MOESM3_ESM.docx]

**Additional File S3.** Predictive performance of diaphragmatic excursion in the emergency department according to the final diagnosis

| Diaphragmatic excursion (cm) | Sensitivity | Specificity | PPV | NPV |
| --- | --- | --- | --- | --- |
| Acute exacerbation of COPD (n=57), AUROC 0.893 (95% CI 0.782–0.999) | | | | |
| ≥2.00 | - | - | - | - |
| 1.60–1.99 | 100.0%  (83.2–100.0) | 48.6%  (31.9–65.6) | 51.3%  (34.8–67.6) | 100.0%  (81.5–100.0) |
| 1.21–1.59 | 100.0%  (83.2–100.0) | 78.4%  (61.8–90.2) | 71.4%  (51.3–86.8) | 100.0%  (88.1–100.0) |
| 0.91–1.20 | 95.0%  (75.1–99.9) | 83.8%  (68.0–93.8) | 76.0%  (54.9–90.6) | 96.9%  (83.8–99.9) |
| ≤0.90 | 45.0%  (23.1–68.5) | 94.6%  (81.8–99.3) | 81.8%  (48.2–97.7) | 76.1%  (61.2–87.4) |
| Without acute exacerbation of COPD (n=257), AUROC 0.830 (95% CI 0.779–0.882) | | | | |
| ≥2.00 | - | - | - | - |
| 1.60–1.99 | 96.8%  (90.9–99.3) | 40.2%  (32.7–48.2) | 47.9%  (40.5–55.3) | 95.7%  (87.8–99.1) |
| 1.21–1.59 | 90.3%  (82.4–95.5) | 58.5%  (50.6–66.2) | 55.3%  (47.0–63.3) | 91.4%  (84.4–96.0) |
| 0.91–1.20 | 79.6%  (69.9–87.2) | 76.8%  (69.6–83.1) | 66.1%  (56.5–74.7) | 86.9%  (80.3–91.9) |
| ≤0.90 | 57.0%  (46.3–67.2) | 87.8%  (81.8–92.4) | 72.6%  (60.9–82.4) | 78.3%  (71.6–84.0) |
| Sepsis (n=149), AUROC 0.792 (95% CI 0.714–0.869) | | | | |
| ≥2.00 | - | - | - | - |
| 1.60–1.99 | 96.1%  (88.9–99.2) | 30.1%  (19.9–42.0) | 58.9%  (49.7–67.6) | 88.0%  (68.8–97.5) |
| 1.21–1.59 | 89.5%  (80.3–95.3) | 52.1%  (40.0–63.9) | 66.0%  (56.0–75.1) | 82.6%  (68.6–92.2) |
| 0.91–1.20 | 78.9%  (68.1–87.5) | 72.6%  (60.9–82.4) | 75.0%  (64.1–84.0) | 76.8%  (65.1–86.1) |
| ≤0.90 | 52.6%  (40.8–64.2) | 86.3%  (76.2–93.2) | 80.0%  (66.3–90.0) | 63.6%  (53.4–73.1) |
| COVID-19 pneumonia (n=33), AUROC 0.893 (95% CI 0.782–0.999) | | | | |
| ≥2.00 | - | - | - | - |
| 1.60–1.99 | 100.0%  (80.5–100.0) | 56.3%  (29.9–80.2) | 70.8%  (48.9–87.4) | 100.0%  (66.4–100.0) |
| 1.21–1.59 | 94.1%  (71.3–99.9) | 75.0%  (47.6–92.7) | 80.0%  (56.3–94.3) | 92.3%  (64.0–99.8) |
| 0.91–1.20 | 76.5%  (50.1–93.2) | 81.3%  (54.4–96.0) | 81.3%  (54.4–96.0) | 76.5%  (50.1–93.2) |
| ≤0.90 | 47.1%  (23.0–72.2) | 87.5%  (61.7–98.4) | 80.0%  (44.4–97.5) | 60.9%  (38.5–80.3) |

AUROC, area under the receiver-operating characteristic curve; CI, confidence interval; COPD, chronic obstructive pulmonary disease; COVID-19, coronavirus disease 2019
